# Neuroendocrine Tumors Have Outgrown the Orphan-Disease Framework: Building an Evidence Base for a Changing Field

**DOI:** 10.3390/cancers18182968

**Published:** 2026-09-14

**Authors:** Aman Chauhan, Jaydira Del Rivero

**Affiliations:** 1 Division of Hematology and Oncology, University of California San Francisco, San Francisco, CA 94143, USA; aman.chauhan@ucsf.edu; 2 Developmental Therapeutics Branch, Center for Cancer Research, National Cancer Institute, National Institutes of Health, Bethesda, MD 20892, USA

For decades, one word shaped the story of neuroendocrine cancer: Rare. It explained why randomized trials were scarce. It justified guidelines built on expert opinion and extrapolation. It excused a grading system that few studies had sufficient numbers to interrogate. Finally, it quietly lowered the standard of evidence we prepare and accept for our patients.

This premise no longer holds.

Contemporary US registry data from 2000 to 2021 show that the incidence of neuroendocrine tumors (NETs) increased substantially over the past two decades. At the same time, prevalence has grown alongside prolonged survival [1]. NETs may still meet some formal definitions of rare cancer. But the orphan-disease framework no longer adequately captures the population burden, nor the strength and maturity we should now expect from the evidence base.

Across two Special Issues of *Cancers* that we had the privilege of co-editing, a group of papers emerged that, read together, make that case more sharply than we anticipated when we wrote the call. Individually they are careful studies, most of them retrospective cohort analysis. Collectively, they describe a field whose clinical practice has often evolved faster than the evidence supporting it.

Consider the natural history of small NETs, which we are only now beginning to measure at scale. Slott and colleagues followed 615 patients over two decades. Median disease-specific survival was 130 months. Among patients who underwent surgery with curative intent, recurrence-free survival rates were 72% at five years and 59% at ten years [2]. The disease did not respect the conventional five-year boundary. Recurrence continued beyond year five, supporting the long surveillance programs already used in neuroendocrine oncology. But the study also challenges one of the tools we use to estimate prognosis. The conventional G1 and G2 categories did not significantly distinguish outcomes. Ki-67 remained informative as a continuous variable rather than a categorical label [2]. That distinction is not merely academic. Every day, categorical grade shapes conversations about prognosis, treatment and surveillance. If the biological signal is continuous, then the boundaries we have drawn may sometimes obscure more than they reveal.

In pancreatic neuroendocrine neoplasms, Møller and colleagues examined another aspect of the same question. Among 51 patients under surveillance for tumors smaller than 2 cm, only one experienced sufficient progression to require resection. In patients who underwent surgery, tumor size, Ki-67 index, and location in the pancreatic head were independently associated with recurrence [3]. For this particular endpoint, these features may carry more information for this particular endpoint than categorical grade alone. The question is no longer simply whether we should observe or intervene. It is whether we are using the most informative measurements to decide.

Then, there is the cost of our vigilance, a cost the filed has been slower to calculate. Iannuzzi and colleagues reviewed 422 surveillance scans performed in 62 patients with completely resected gastroenteropancreatic NETs. The mean cumulative effective dose of 56.05 mSv. Extrapolated across ten years of imaging, exposure approached 106 millisieverts (mSv) [4]. The authors noted that this cumulative exposure lies in a range associated with secondary malignancies. Placed beside the long-term recurrence data, the dilemma becomes unavoidable. Late recurrence is real. Long surveillance may be justified, but surveillance is not biologically neutral. Repeated ionizing imaging may introduce a different form of risk, one that accumulates silently with every scan. For years, surveillance recommendations have emphasized the importance of detecting recurrence. Future guidelines should also account for cumulative radiation exposure and consider strategies that balance both risks.

Some diseases remain genuinely and profoundly rare. Piórek and colleagues assembled a cohort of 19 patients with thymic neuroendocrine neoplasms treated at a single comprehensive cancer center over 25 years—a quarter of a century. Median overall survival was 127 months, but that number concealed the difference that matters the most: 170 months for patients treated with curative intent, compared with 33 months for those receiving palliative therapy. Even among patients undergoing radical surgery, an R0 resection was achieved in only 25% [5]. This is where rarity remains most consequential in neuroendocrine oncology: in exceptional subtypes for which decades of experience at a major center produce a cohort smaller than a single phase I trial.

Here, uncertainty is not an excuse. It is an unavoidable condition and one that underscores the value of collaboration across institutions, registries, and borders. The search for better biomarkers reflects a field still learning how to turn biological signals into clinically useful tools. Bocian-Jastrzębska and colleagues examined leptin, adiponectin, and their ratio in 83 patients with pancreatic neuroendocrine neoplasms and 39 controls. They identified associations with tumor grade, proliferative index, and metastatic status [6]. The findings represent a signal, not yet a test. That distinction matters. Progress depends not only on discovering new associations, but also on resisting the temptation to claim more certainty than the data can support. We believe this work is worth publishing precisely as it is: promising, informative, and appropriately preliminary. Perhaps nowhere is uncertainty more visible than in higher-grade, well-differentiated disease.

Patients with higher G2 tumors (defined by a Ki-67 index of 10–20%), G3 tumors (Ki-67 index 20–55%), and gastroenteropancreatic NETs, still have a clearly established treatment sequence. Much of current practice is extrapolated from lower-grade disease or built on small retrospective series [7]. In our review, developed with colleagues, we recommend baseline somatostatin receptor PET imaging for patients with well-differentiated higher G2 or G3 disease. For receptor-positive patients who are clinically stable, peptide receptor radionuclide therapy may be considered as a first-line option. Chemotherapy may be better suited to patients who require rapid tumor reduction, including those in visceral crisis, or when somatostatin receptor imaging or PRRT is unavailable [7]. Reasonable clinicians will disagree with parts of this approach. But the deeper issue is not a disagreement. It is that too many of these decisions are still influenced by institutional habit, local access and individual experience rather than prospective comparative data.

Across these Special Issues, the most important studies are not necessarily those that confirm established practice. They are the ones that make practice more difficult: A grading boundary that may blur prognosis rather than clarify it; a surveillance strategy that may detect recurrence while also creating cumulative radiation exposure; an exceptionally rare malignancy in which complete surgical resection remains difficult to achieve, even at an expert center. These findings are not discouraging. They are evidence of a field coming of age. Neuroendocrine oncology may finally have enough patients, enough years of follow-up, and enough accumulated experience to examine the assumptions on which modern care has been built.

The task ahead is not to persuade anyone that neuroendocrine tumors matter. Contemporary incidence and prevalence data have made the burden impossible to ignore. The task now is to ensure that the evidence base evolves alongside that growing burden. We need to move beyond designing our trials, writing our guidelines, and planning lifelong surveillance as though only a handful of people were living with this disease. “Rare” can no longer serve as a sufficient explanation for limited evidence. Instead, rarity should motivate broader collaboration, more rigorous measurement, and continued efforts to strengthen the science, because these patients are no longer too few to study, nor were they ever too few in number to matter.
